# A COMMENTARY ON METHODOLOGICAL ISSUES IN RESEARCH ON REHABILITATION NEEDS AFTER MODERATE-TO-SEVERE TRAUMATIC INJURY: LESSONS LEARNED FROM QUANTITATIVE AND QUALITATIVE STUDIES IN NORWAY

**DOI:** 10.2340/jrm.v58.46422

**Published:** 2026-10-06

**Authors:** Emilie Isager HOWE, Mari S. RASMUSSEN, Håkon Ø. MOKSNES, Christoph SCHÄFER, Helene L. SØBERG, Cecilie RØE, Torgeir HELLSTRØM, Nada ANDELIC

**Affiliations:** 1 Department of Physical Medicine and Rehabilitation, Oslo University Hospital, Oslo; 2 Faculty of Health Sciences, Oslo Metropolitan University, Oslo; 3 Institue of Health and Society, Faculty of Medicine, University of Oslo, Oslo; 4 Department of Physical Medicine and Rehabilitation, University Hospital of North Norway, Tromsø; 5 Department of Clinical Medicine, Faculty of Health Sciences, UiT-The Arctic University of Norway, Tromsø; 6 Institute of Clinical Medicine, Faculty of Medicine, University of Oslo, Oslo, Norway

Access to rehabilitation is fundamental to ensuring continued participation and quality of life for individuals with functional limitations. The overarching goal of any rehabilitation intervention is to promote independence, well-being, and participation in accordance with a person’s life situation and goals. Conversely, unmet rehabilitation needs can limit activities, restrict participation, deteriorate health, reduce autonomy, and diminish both quality of life and life satisfaction (1).

Although broad categories of unmet psychological, cognitive, and vocational rehabilitation needs have been identified after specific injuries (2), the rehabilitation needs of people with moderate-to-severe traumatic injuries remain insufficiently understood (3). Few studies have examined how rehabilitation service users articulate their needs or perceive the services they receive. Moreover, different stakeholders, patients, family members, rehabilitation professionals, and decision-makers may define and evaluate rehabilitation needs in different ways. Exploring and comparing these perspectives is essential for developing a comprehensive understanding and for ensuring that services align with users’ priorities. Ultimately, such insights may promote shared decision-making and person-centred care.

Methodologically, research on rehabilitation needs after trauma faces several challenges. These include the selection of study populations and follow-up periods, the choice and validity of outcome measures, operational definition of rehabilitation needs, and the integration of quantitative and qualitative data.

The aim of this paper is to offer foundational guidance for researching rehabilitation needs after trauma. Drawing on our experiences with both quantitative and qualitative studies, we highlight key methodological challenges and describe strategies to address them.

## OVERVIEW OF STUDIES

We conducted several quantitative studies with survivors of moderate-to-severe trauma to explore rehabilitation needs, utilization of rehabilitation services, prevalence of unmet needs, and functional outcomes within the first year after injury (4–8). Approximately half of the patients experienced persistent disability. Pre-injury comorbidities, various aspects of injury severity, and estimated rehabilitation needs emerged as important predictors of overall functional outcomes (5, 6).

Acute rehabilitation in the intensive care unit was documented for most patients; however, recommended direct transfers from acute wards to specialized rehabilitation units were uncommon (4). Although needs for subacute inpatient rehabilitation were largely met, there was a high prevalence of unmet needs for community-based health and rehabilitation services during the first-year post-injury. The greatest proportion of unmet needs concerned rehabilitation services, followed by social and family support (7).

In addition, a qualitative study explored experiences and perceptions of rehabilitation needs and services across different levels of care by using Bradshaw’s taxonomy of need as a theoretical framework (9), distinguishing between normative needs (defined by professional standards or expert opinion) and felt (service users’ perceptions) and expressed needs (felt need translated into demand). The study involved in-depth individual and focus-group interviews with individuals who had sustained traumatic injuries, their family members, and rehabilitation professionals (10). Participants described how the long-term consequences of their injuries extended beyond the temporal scope of available rehabilitation services, resulting in unmet needs after formal support had ended. Many participants reported feeling unprepared for the psychological reactions that emerged following their injuries. They described the injury event itself as emotionally traumatic and felt insufficiently prepared for the subsequent emotional and psychological responses, which were often delayed in onset. Participants expressed a need for continuous, individualized rehabilitation services that extended beyond hospital discharge. Rehabilitation professionals similarly identified continuity of care and multidisciplinary collaboration as essential components of high-quality rehabilitation. They also emphasized the need to strengthen community-based rehabilitation services for individuals who sustain traumatic injuries (9).

## METHODOLOGICAL ISSUES

We conducted an observational prospective cohort study to examine how exposure affects outcomes over time in a group of patients with moderate-to-severe trauma (8). This design was deemed appropriate for including a large and representative sample of patients admitted to two trauma centres. The use of the New Injury Severity score (NISS) (11) and the duration of hospital stay as inclusion criteria provides a clear-cut description of the population that can be reproduced in other studies. The rationale for focusing on moderate-to-severe trauma (NISS > 9) was based on a recommendation from the National Institute for Health and Care Excellence that patients with an Injury Severity Score (ISS) > 9 in a trauma centre should be assessed for rehabilitation needs and receive a rehabilitation prescription, if necessary. However, we used the NISS scores as its calculation method allows for a more accurate rating of the severity of multiple injuries and identifies a higher proportion of individuals with major trauma (11).

A multicentre approach, involving 2 trauma centres, provided a catchment area representing more than 60% of the general trauma centre population in Norway and allowed us to explore health services and outcomes across demographic and regional differences. Ensuring a high inclusion rate among eligible patients is crucial for achieving representativeness. According to Teague et al., retention can be improved by offering participants alternative methods of data collection (e.g., completing interviews by telephone) (12). We adopted this follow-up strategy and achieved follow-up rates ranging from 70% to 86%, indicating that both the inclusion of eligible patients and follow-up rates were acceptable in this study.

Follow-up assessments were conducted at 6 and 12 months post-injury to capture the most critical recovery period. It was assumed that most patients would have completed subacute inpatient rehabilitation within 6 months. A follow-up period longer than 12 months was not feasible due to the project’s timeframe. A limitation is the lack of more extensive pre-injury data on participants’ health and functioning, which may influence post-injury outcomes. Retrospective data would be required to address this gap. Furthermore, the effects of specific treatments or service provision cannot be established in a design that does not include control groups or randomization.

Within this context, qualitative data may supplement quantitative findings by providing in-depth insights into healthcare and rehabilitation needs, supporting the interpretation of results, evaluating the feasibility and value of rehabilitation services, and identifying barriers and facilitators to implementation. In our study, qualitative interviews generated rich data that can contribute to the development of rehabilitation services and inform future research projects.

Loss due to inclusion and follow-up can introduce bias in cohort studies. The present study has a relatively high inclusion rate, but we cannot rule out the possibility that those who did not participate or were not included may differ systematically from the included participants. In this regard, although we cannot collect information regarding causes for not participating, it is known that marginal groups may have lower inclusion and follow-up rates in research. Despite this concern, the number of potential participants lost to inclusion or follow-up was low and did not show large differences compared with those who attended, except for slightly lower age for those lost to follow-up. We note that this study was conducted during the COVID-19 pandemic, which may have affected hospital admission patterns. Nevertheless, the study population was restricted to patients with a NISS > 9, who during this period would have been admitted to and treated within trauma hospitals rather than outside the trauma care system (6).

## OUTCOME MEASUREMENTS

While the body of research following trauma would benefit from greater consistency in measuring common unmet rehabilitation needs, researchers must carefully consider each outcome in relation to the study’s aims, the theoretical framework employed, and participants’ response burden.

The main outcomes in our quantitative research were the Rehabilitation Complexity Scale – Trauma (RCS–E Trauma) to assess subacute inpatient rehabilitation needs (13, 14) and the Needs and Provision Complexity Scale (NPCS) to evaluate community-based healthcare, rehabilitation, and social support (14). The NPCS has been validated in a Norwegian population with traumatic brain injury (TBI) and subarachnoid haemorrhage (SAH) (15). The RCS-E has been translated into Norwegian; however, its psychometric properties have not yet been validated in a Norwegian context.

Data were collected via telephone interviews to minimize missing data by reaching patients across a wider geographic area, including elderly populations and individuals with complex health concerns. However, it is well known that an interviewer’s tone, pace, and style can inadvertently bias participants’ responses. To address this, we provided thorough interviewer training. Nevertheless, this approach was time-intensive, particularly when using instruments with complex questions that could be difficult to understand when administered orally, thereby introducing the possibility of respondent error. Consequently, some participants preferred to complete the instruments electronically or using paper-and-pencil formats, and these preferences were accommodated when feasible.

The qualitative research approach focused on gaining in-depth insights through an interview guide with open-ended questions, allowing for rich narratives and a nuanced understanding of participants’ perspectives. For this component, we used a mix of face-to-face and telephone interviews, balancing the richness and non-verbal cues afforded by in-person conversations with the convenience and broader reach of telephone contact, thereby supporting comprehensive and high-quality data collection.

### Limitations

The heterogeneity of rehabilitation outcome measures used in trauma research remains a significant limitation for systematic reviews, restricting the ability to draw firm conclusions about the most appropriate tools for assessing rehabilitation needs. In the quantitative component of our study, we employed the RCS-E Trauma and NPCS instruments. These instruments were a natural choice because alternative measures are scarce, and previous studies have recommended them as suitable tools. To facilitate comparison between unmet needs calculated with the RCS–E Trauma and the NPCS, we applied the same scoring method to both scales (6). This approach has not been used in prior research involving the RCS–E Trauma and therefore warrants validation in future studies.

Rehabilitation specialists estimated rehabilitation needs based on injury characteristics at baseline and patient-reported impairments at the 6-month follow-up. However, healthcare professionals and rehabilitation providers in community settings may have different perspectives on patients’ rehabilitation needs. Furthermore, neither the availability of rehabilitation services nor the effectiveness of specific interventions in addressing identified needs was considered. Finally, unmet needs reported by patients at 6 and 12 months may reflect not only a lack of available or effective services, but also limited utilization of, or adherence to, the services provided. As a result, rehabilitation needs may have been overestimated, possibly due to an underestimation of functional improvements between 6 and 12 months post-injury. Other limitations lie in the conceptual challenges of defining and rationalizing rehabilitation needs – and, in turn, understanding how to fulfil them – as reflected in the lack of consensus on the concept itself. The notion of rehabilitation needs and functional outcomes may hold different meanings for various stakeholders involved in the processes we aim to study and improve. These stakeholders include patients, caregivers, healthcare professionals, policymakers, decision-makers, and, more broadly, society. As a result, estimating an individual patient’s needs can be complex, as they are likely influenced, at least in part, by often unmeasured factors such as personality, communication style, and resilience. An additional consideration is that several well-established risk factors for poor outcomes have been identified, including premorbid health conditions, sociodemographic factors such as education and employment, availability of social support and other resources, and injury-related factors. These factors may influence both recovery and the ability to access and benefit from rehabilitation. Consequently, observed rehabilitation needs may partly reflect pre-existing health and social inequalities rather than the consequences of trauma alone. These factors could be systematically assessed for each patient to develop an individual risk profile, which may help estimate rehabilitation needs more accurately and support the design of more tailored rehabilitation pathways. However, identifying and estimating individual needs is only part of the challenge; ensuring that rehabilitation services have the capacity and flexibility to respond to these needs remains equally important.

In our qualitative study exploring different perspectives on rehabilitation needs, patients and rehabilitation professionals represented both urban and rural geographic settings. However, the rehabilitation professionals did not encompass all occupational groups, and women employed in specialist healthcare constituted approximately two-thirds of the participants. In addition, the researchers conducting these studies are themselves rehabilitation professionals, and the findings should be interpreted considering this professional background. We acknowledge that our professional perspectives may have influenced the interpretation of the data and the identification of themes, potentially leading to certain aspects being unintentionally emphasized or overlooked. At the same time, our extensive experience in rehabilitation may have enhanced our ability to capture and interpret nuances in the participants’ accounts.

Taken together, rehabilitation needs after trauma are shaped by individual factors, as discussed earlier, whereas whether these needs are met also depends on the wider health system. As recovery following major trauma may extend over months or years, effective rehabilitation requires continuity and coordination across acute care, specialized rehabilitation, primary and community-based services, as well as mental health, social, and vocational services. System-level factors, including workforce capacity, geographical availability of services, referral pathways, and coordination across levels of care, may influence access to rehabilitation and contribute to inequalities in service provision. Persistent unmet needs may therefore reflect not only individual impairments and personal and social circumstances, but also limitations in the availability, accessibility, and coordination of rehabilitation services. As these system-level factors were not systematically assessed in the present project, they should be considered when interpreting our results.

### Conclusion

Access to rehabilitation is a key determinant of improved functioning and health following trauma, and rehabilitation research generates new knowledge to inform strategies that protect and promote well-being. However, the quality and potential impact of studies on rehabilitation needs and service delivery after trauma depend on methodological rigour. This paper highlights common methodological challenges in this field and presents lessons learned from several quantitative and qualitative studies on rehabilitation needs following trauma.

By describing the challenges encountered and the approaches used to address them, we aim to support researchers undertaking similar studies. Greater methodological discourse on the study of rehabilitation needs will not only enhance research quality and strengthen study designs but also inform the implementation of programmes that bridge the gap between identified needs and access to and utilization of rehabilitation services.

## References

[1] Kamenov K, Mills JA, Chatterji S, Cieza A. Needs and unmet needs for rehabilitation services: a scoping review. Disabil Rehabil 2019; 41: 1227–1237. 10.1080/09638288.2017.1422036 29303004

[2] Andelic N, Soberg HL, Berntsen S, Sigurdardottir S, Roe C. Self-perceived health care needs and delivery of health care services 5 years after moderate-to-severe traumatic brain injury. PM R 2014; 6: 1013–1021. 10.1016/j.pmrj.2014.05.005 24844444

[3] Jones SM, West C, Rappoport J, Akhtar K. Rehabilitation outcomes based on service provision and geographical location for patients with multiple trauma: a mixed-method systematic review. Injury 2023; 54: 887–895. 10.1016/j.injury.2023.01.034 36801069

[4] Schäfer C, Mosknes H, Rasmussen MS, Hellstrøm T, Lundgaard Soberg H, Røise O, et al. Adherence to Guidelines for Acute Rehabilitation in the Norwegian Trauma Plan. J Rehabil Med 2023; 55: jrm6552. 10.2340/jrm.v55.6552 PMC10312324 37366570

[5] Moksnes H, Schäfer C, Rasmussen MS, Soberg HL, Røise O, Anke A, et al. Functional outcomes at 6 and 12 months post-injury in a trauma centre population with moderate-to-severe traumatic injuries. J Clin Med 2023; 12: 5300. 10.3390/jcm12165300 PMC10455533 37629342

[6] Moksnes HO, Andelic N, Schafer C, Anke A, Soberg HL, Roe C, et al. Unmet rehabilitation needs in the first 6 months post-injury in a trauma centre population with moderate-to-severe traumatic injuries. J Rehabil Med 2024; 56: jrm40078. 10.2340/jrm.v56.40078 PMC11151492 38803207

[7] Andelic N, Moksnes H, Rasmussen MS, Schafer C, Hellstrom T, Howe EI, et al. Needs for community-based rehabilitation services and support 12 months after moderate and severe physical traumatic injuries: a brief report. Am J Phys Med Rehabil 2024; 103: 740–744. 10.1097/PHM.0000000000002492 38662737

[8] Soberg HL, Moksnes HO, Anke A, Roise O, Roe C, Aas E, et al. Rehabilitation needs, service provision, and costs in the first year following traumatic injuries: protocol for a prospective cohort study. JMIR Res Protoc 2021; 10: e25980. 10.2196/25980 PMC8082380 33688841

[9] Howe EI, Soberg HL, Roe C, Lovstad M, Andelic N. “What standard should we set?”: a qualitative study of rehabilitation professionals’ perspectives on rehabilitation needs after traumatic injuries. J Rehabil Med 2025; 57: jrm44087. 10.2340/jrm.v57.44087 PMC12587454 41168954

[10] Howe EI. Trauma rehabilitation: user experiences and unmet needs: OSF platform; 2021. Available from https://osf.io/2hra8/overview

[11] Lavoie A, Moore L, LeSage N, Liberman M, Sampalis JS. The New Injury Severity Score: a more accurate predictor of in-hospital mortality than the Injury Severity Score. J Trauma 2004; 56: 1312–1320. 10.1097/01.TA.0000075342.36072.EF 15211142

[12] Teague S, Youssef GJ, Macdonald JA, Sciberras E, Shatte A, Fuller-Tyszkiewicz M, et al. Retention strategies in longitudinal cohort studies: a systematic review and meta-analysis. BMC Med Res Methodol 2018; 18: 151. 10.1186/s12874-018-0586-7 PMC6258319 30477443

[13] Turner-Stokes L, Scott H, Williams H, Siegert R. The Rehabilitation Complexity Scale – extended version: detection of patients with highly complex needs. Disabil Rehabil 2012; 34: 715–720. 10.3109/09638288.2011.615880 22115200

[14] Turner-Stokes L, McCrone P, Jackson DM, Siegert RJ. The Needs and Provision Complexity Scale: a multicentre prospective cohort analysis of met and unmet needs and their cost implications for patients with complex neurological disability. BMJ Open 2013; 3: e002353. 10.1136/bmjopen-2012-002353 PMC3586084 23435796

[15] Forslund MV, Borgen IMH, Karic T, Kleffelgard I, Hauger SL, Lovstad M, et al. Validity of the Norwegian Version of the Needs and Provision Complexity Scale (NPCS) in patients with traumatic brain injury and atraumatic subarachnoid hemorrhage. J Clin Med 2024; 13: 752. 10.3390/jcm13030752 PMC10856215 38337446

